# Dr. John F. Cleveland

**Published:** 1918-07

**Authors:** 


### OBITUARY

**Readers are requested to report promptly the death of all physicians in Western New York, or former residents of this region, or graduates of any medical school in Western New York, and to notify the families of the deceased of our desire to publish adequate Obituary notices.**

**Dr. John F. Cleveland,** Le Roy, Bellevue Hospital Medical College, New York, 1865. Died April 16, age 86. He was a veteran of the Civil War.

